# Acceptance of a Third Dose of COVID-19 Vaccine and Associated Factors in China Based on Health Belief Model: A National Cross-Sectional Study

**DOI:** 10.3390/vaccines10010089

**Published:** 2022-01-07

**Authors:** Chenyuan Qin, Ruitong Wang, Liyuan Tao, Min Liu, Jue Liu

**Affiliations:** 1 Department of Epidemiology and Biostatistics, School of Public Health, Peking University, Beijing 100191, China; qincy@bjmu.edu.cn (C.Q.); wangruitong@pku.edu.cn (R.W.); liumin@bjmu.edu.cn (M.L.); 2 Research Center of Clinical Epidemiology, Peking University Third Hospital, Beijing 100083, China; tendytly@163.com; 3 Institute for Global Health and Development, Peking University, Beijing 100871, China; 4 National Health Commission Key Laboratory of Reproductive Health, Peking University, Beijing 100191, China

**Keywords:** COVID-19, vaccination, acceptance, third dose, associated factors

## Abstract

COVID-19 infections are returning to many countries because of the emergence of variants or declining antibody levels provided by vaccines. An additional dose of vaccination is recommended to be a considerable supplementary intervention. We aim to explore public acceptance of the third dose of the COVID-19 vaccine and related influencing factors in China. This nationwide cross-sectional study was conducted in the general population among 31 provinces in November, 2021. We collected information on basic characteristics, vaccination knowledge and attitudes, and vaccine-related health beliefs of the participants. Univariable and multivariable logistic regression models were used to assess factors associated with the acceptance of a third COVID-19 vaccine. A total of 93.7% (95% CI: 92.9–94.6%) of 3119 Chinese residents were willing to receive a third dose of the COVID-19 vaccine. Individuals with low level of perceived susceptibility, perceived benefit, cues to action cues, and high level of perceived barriers, old age, low educational level, low monthly household income, and low knowledge score on COVID-19 were less likely to have the acceptance of a third dose of COVID-19 (all *p* < 0.05). In the multivariable logistic regression model, acceptance of the third dose of COVID-19 vaccine was mainly related to previous vaccination history [Sinopharm BBIP (aOR = 6.55, 95% CI 3.30–12.98), Sinovac (aOR = 5.22, 95% CI:2.72–10.02), Convidecia (aOR = 5.80, 95% CI: 2.04–16.48)], high level of perceived susceptibility (aOR = 2.48, 95% CI: 1.48–4.31) and high level of action cues (aOR = 23.66, 95% CI: 9.97–56.23). Overall, residents in China showed a high willingness to accept the third dose of COVID-19 vaccines, which can help vaccine manufacturers in China to manage the vaccine production and distribution for the huge domestic and international vaccine demand. Relevant institutions could increase people’s willingness to booster shots by increasing initial COVID-19 vaccination rates, public’s perception of COVID-19 susceptibility and cues to action through various strategies and channels. Meanwhile, it also has certain reference significance for other countries to formulate vaccine promotion strategies.

## 1. Introduction

COVID-19 has raged around the world for nearly two years since it was defined as a global pandemic by the World Health Organization (WHO) after a multiparty assessment on 11 March 2020 [1]. As of 24 December 2021, more than 278 million people have experienced or are currently experiencing this acute respiratory infection, and nearly 5.4 million people have died as a result [2]. There is no denying that vaccination is the most cost-effective public health intervention to prevent and control COVID-19 in the absence of effective treatments. Updated on 24 December 2021, 23 of 333 vaccine candidates have been put into production [3]. The latest data showed that China has reported that a total of 2.75 billion doses of COVID-19 vaccine have been injected for free [4].

Despite the great success of previous vaccination campaigns, COVID-19 infections are making a comeback in many countries [5,6]. Several studies at home and abroad have suggested that this may be related to the fact that the current infection is mainly caused by variants (particularly the delta variant) or the immunity provided by vaccines is gradually weakened over time [7,8]. The standard way to overcome weakened immunity is to give an extra dose of vaccine—a third dose (or a booster dose) [5]. Therefore, a third dose of vaccination on top of the original complete vaccination is seen as a considerable supplementary measure [9]. To improve the immunity of the local population, enhance resistance to variants and maintain the stability of social order, countries have gradually strengthened vaccination, mainly including China, the United States, the United Kingdom, and Israel, etc. [10]. In addition to homologous vaccines, heterologous vaccines are also used in other countries, such as the United States, the United Kingdom, Chile and so on [10]. As of 24 December 2021, 477.27 million booster shots have been administered globally (6.06 doses per 100 people) [10].

As a public health threat, vaccine hesitancy has been cited as one of the possible causes of declining vaccination coverage and increased outbreaks of vaccine-preventable diseases [11,12]. Acceptance of the third dose of COVID-19 vaccine varied among different countries. For healthcare workers in the United States, the overall acceptance rate for a booster vaccination was 83.6% [13]. In addition, it was high (71.3%) among healthcare workers in Czechia, while 12.2% remained undecided and 16.6% opposed [14]. In Poland, 71% the adults expressed willingness to receive the booster dose [15]. Sugawara et al. revealed in 2021 that 89.1% of Japanese medical students were positive about a booster vaccination [16]. The main reasons against receiving included side effects after previous doses, a perception that further vaccination was not necessary, and the distrust of regulatory agencies (45.3%), government (48.6%) and drug companies (50%) [13,15].

To our knowledge, research on public uptake of booster doses of COVID-19 vaccine and related factors still leaves a large gap, particularly in China. Therefore, based on the health belief model (HBM), we assessed the Chinese public’s acceptance and influencing factors of a third dose of COVID-19 vaccine in the context of the pandemic. Our results could provide feasible inspiration for targeted measures and policy recommendations for universal vaccination coverage of the third dose of COVID-19 vaccine. Further translation of vaccination intentions into vaccination behaviors through health education and policy advocacy is supposed to be an ultimate expectation.

## 2. Methods

### 2.1. Study Design and Participants

We conducted a nationwide online survey of people’s willingness to receive the third dose of COVID-19 vaccine from 12–17 November 2021. The data were collected using a questionnaire platform (Wen Juan Xing). As the largest online survey platform in China, Wen Juan Xing has nearly 3 million registered members with clear personal information and diverse social and economic backgrounds, so it could collect representative samples of the Chinese population. Participants aged 18 or above were the target subjects in this study. We randomly allocated the questionnaires among 31 provinces in the platform (Supplementary Table S1), based on the population proportion of 31 provinces in the China Statistical Yearbook 2021. A total of 3119 valid questionnaires were collected.

### 2.2. Data Collection

Based on our previous COVID-19 vaccination willingness questionnaire [17,18], we specifically focused on the acceptance of the third dose of COVID-19 vaccine and its associated factors with health beliefs in this study. we investigated information including basic personal characteristics, attitudes towards a third dose of COVID-19 vaccine, knowledge of COVID-19 infection and vaccines, and vaccine-related health beliefs. Basic characteristics mainly includes sociodemographic characteristics, health status and COVID-19 vaccination history. sociodemographic characteristics included gender, age, educational background, occupation, and economic level. health status included chronic disease history and COVID-19 vaccination history. “Do you have any chronic disease like cardiovascular disease, cancer, diabetes, chronic respiratory disease or others?” was used to collect the participants’ chronic medical history. Given that the third dose is already available in China to eligible people (those who have completed two full doses for more than six months), the vaccination history in this study included an inquiry into the status of three injections. According to our previous studies [18,19], knowledge of COVID-19 was investigated, including the sources of infection, common symptoms, prevention measures, high-risk groups, susceptible population, vaccine effectiveness, etc. Under prespecified circumstances, each correct choice got 1 score, while 0 represented the other response. The range of total score on knowledge of COVID-19 and COVID-19 vaccine is 0~16 and 0~3, respectively, then they were divided into three degrees by tertials. Specifically, for the score of COVID-19 knowledge, 0~4 represented low degree, 5~11 represented moderate degree and 12~16 representing high degree. The knowledge score of the COVID-19 vaccine was also divided into three grades from low to high: 0, 1~2, and 3.

### 2.3. Acceptance of a Third Dose of COVID-19 Vaccine

We assessed the acceptance by asking “Would you be willing to receive a third dose of COVID-19 vaccine?” The acceptance rate of a third dose of COVID-19 vaccine was defined as the proportion of participants who answered “yes” to all participants in this study. If the answer is the “No” or “Not Sure”, the reason for their hesitation to vaccinate will be further explored.

### 2.4. Health Beliefs on COVID-19 and COVID-19 Vaccine

Based on the principle of HBM and previous literature [17,18,20], we set 13 questions from five HBM dimensions, which were answered by all participants. HBM hypothesizes that susceptibility to disease, severe outcome, beneficial behavior, and few obstacles are positive factors that promote individuals to adopt disease-prevention behaviors, such as vaccination [21]. Vaccine-related information from doctors, family members, community workers and others can also influence individual behavior. The questionnaire mainly included perceptions of oneself and family members’ COVID-19 susceptibility and infection severity (2 items each), perceptions of barriers and benefits to COVID-19 vaccination, and cues to action (3 items each).

All questions were answered in accordance with “very Concerned/Agree/True”, “Concerned/Not Sure/Unclear” and “Not Concerned/Disagree/False”, which were marked as 3 points, 2 points and 1 point, respectively. After calculating the total scores of each of the 5 HBM dimensions, the susceptibility perception and severity perception scores were graded in descending order of 2~3, 4~5, and 6. The other dimensions were set as “Low”, “Moderate” and “High” with 3~4 points, 5~7 points and 8~9 points, respectively.

### 2.5. Data Analysis

We used frequency and percentage to summarize the characteristics of the recruited population (sociodemographic characteristics, health status, knowledge factors and health beliefs). The proportion and 95% CI of participants’ willingness to receive a third dose of COVID-19 vaccine of total population and different characteristics groups were calculated. Pearson χ^2^ test was used to compare the differences on acceptance of a third dose of COVID-19 vaccine under certain characteristics.

Univariable and multivariable logistic regression models were both performed to explore the associations between individual factors and the acceptance of a third dose of COVID-19 vaccine. We selected the significant variables to be part of the multivariable model based the Pearson’s χ^2^-test. Individual variables included sociodemographic characteristics, health status, relevant knowledge scores and health belief scores. The crude odds ratio (cOR), adjusted odds ratio (aOR), and 95% CI for each variable were calculated. Hosmer and Lemeshow Test was used to assess the goodness of model fitting.

Data analysis was performed by R 4.0.3 (AT&T Bell Laboratories, Auckland, New Zealand) and SPSS 26.0 (International Business Machines Corporation, New York, NY, USA), a *p* value less than 0.05 was indicated statistical significance.

## 3. Results

### 3.1. Characteristics of the Participants

A total of 3119 eligible participants were recruited for this study (Table 1). More than 40% lived in eastern China, 48.8% were female, 61.2% were under 30 years old, and 80.9% had a bachelor’s degree or above. The vast majority (92.4%) had no history of chronic diseases, and 3025 (97.0%) had received at least one dose of the COVID-19 vaccine.

### 3.2. Acceptance of a Third Dose of COVID-19 Vaccine

Of all participants recruited, 2923 (93.7%, 95% CI: 92.9–94.6%) were willing to receive a third dose of COVID-19 vaccine (Table 1). There was no significant difference in vaccine acceptance among different regions in China (*p* = 0.06). The acceptance rate showed a unimodal change with increasing age, and the highest acceptance rate was 94.6% (95% CI: 93.4–95.6%) in the 21–30 years aged group. The vaccination acceptance rate in the >50 years aged group decreased to 81.7% (95% CI: 72.3–88.9%). Uptake of the third dose of COVID-19 vaccine continued to increase along with socioeconomic status (*p* < 0.01). People with higher educational level, working in a state agency or public institution, or received at least one dose of COVID-19 vaccine were more likely to vaccinate a third dose (all *p* < 0.01). As individual infection and COVID-19 vaccine knowledge scores increased, ultimately more than 95% of participants expressed willingness to receive the third dose of COVID-19 vaccine.

### 3.3. Comparison of the Acceptance of a Third Dose of COVID-19 Vaccine by Dimensions of Health Belief Model

**Figure 1:**
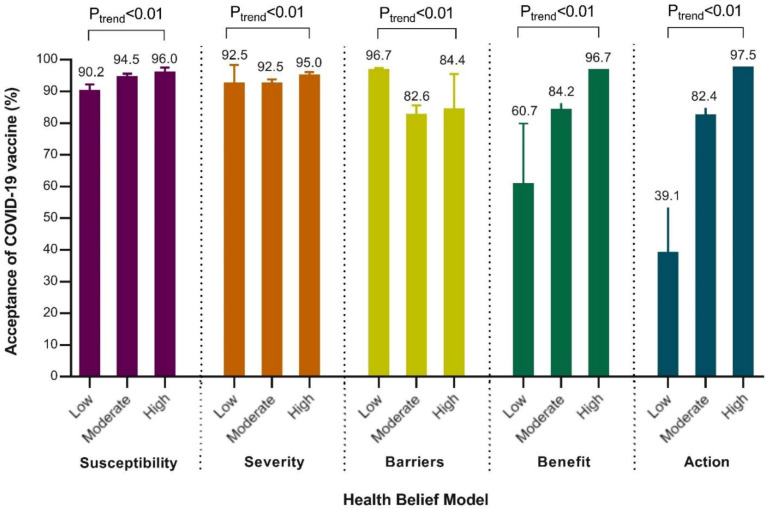
The acceptance of a third dose of COVID-19 vaccine by five dimensions of the health beliefs model (n = 3119).

Participants who were concerned that they or a family member contracted COVID-19 were more willing to receive a third dose of COVID-19 vaccine than those who were not concerned (*p* < 0.05) (Table 2). On the “Perceived Severity” dimension, participants who perceived that they would have an adverse impact on their family members’ health after infection were more likely to be vaccinated (94.3%, 95% CI 93.4–95.1%). However, while those who agreed that their infection would cause severe illness were more likely to accept the third dose of vaccine than those who disagreed, the difference was not statistically significant (*p* = 0.06). Participants who disagreed that the safety and efficacy of current COVID-19 vaccines remained to be proven were more likely to be vaccinated than those who agreed (*p* < 0.05). Based on the analysis of five dimensions of HBM, it was found that the higher the perception of “susceptibility”, “severity”, “benefit” and “cues to action”, the higher the acceptance rate of the third dose of COVID-19 vaccine (*P*_trend_ < 0.01) (Figure 1). In addition, participants were more willing to accept when perceived barriers at the “Low” level than at the other two levels (*P*_trend_ < 0.01).

### 3.4. Factors Related with a Third Dose of COVID-19 Vaccine Acceptance

Individuals with high level of perceived susceptibility (cOR = 2.60, 95% CI: 1.63–4.13), perceived benefit (cOR = 19.17, 95% CI: 8.70–42.26), cues to action cues (cOR = 61.28, 95% CI: 32.17–116.82) and low level of perceived barriers (cOR = 3.45, 95% CI: 1.57–7.57) were more likely to have higher acceptance of a third dose of COVID-19 in the univariable models. (Table 3). In addition, age, educational background, occupation, history of COVID-19 vaccination, knowledge of COVID-19 and vaccine were also influential factors.

Multivariable logistic regression displayed that the major factors related to the acceptance of the third dose of COVID-19 vaccine were vaccination history [Sinopharm BBIP (aOR = 6.55, 95% CI 3.30–12.98), Sinovac (aOR = 5.22, 95% CI: 2.72–10.02), Convidecia (aOR = 5.80, 95% CI: 2.04–16.48)], high level perceived susceptibility (aOR = 2.48, 95% CI: 1.48–4.31) and high level of action cues (aOR = 23.66, 95% CI: 9.97–56.23). Additionally, it is worth noting that compared to civil servants, employees of enterprises (aOR = 0.21, 95% CI: 0.05–0.98), individual households (aOR = 0.11, 95% CI: 0.02–0.53), and other occupations (aOR = 0.10, 95% CI: 0.02–0.57) had lower vaccination willingness.

### 3.5. Reasons for Hesitation to Receive a Third Dose of COVID-19 Vaccine

**Figure 2:**
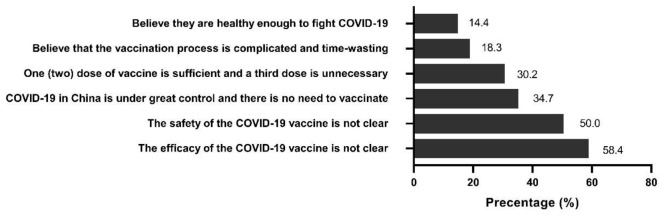
Reasons for responding “No” or “Not sure” regarding willingness to accept a third dose of COVID-19 vaccine (n = 196).

Among the 196 participants who expressed hesitation to receive a third dose of COVID-19 vaccine (answering no or not sure), 58.4% and 50.0% of them hesitated due to uncertainty about the efficacy and safety of the vaccine, respectively (Figure 2). About one-third of the participants believed that a third dose is not necessary because those one or two doses of the COVID-19 vaccine were sufficient to avoid infection. Perceptions that the vaccination process is cumbersome and time-wasting or that they were at low risk of infection were also reasons for hesitancy.

## 4. Discussion

To our knowledge, this is the first nationwide cross-sectional study to assess public acceptance of receiving a third dose of COVID-19 vaccine and its influencing factors, using a random sample of the national population in China. In the context of wide injection of the initial two doses, we found that the acceptance rate of the third dose among mainland China was 93.7% (95% CI 92.9–94.6%). The main factors related to the vaccine acceptance rate were civil servant occupation, vaccination history, high level of perceived susceptibility and high level of action cues. Concerns about the safety and effectiveness of COVID-19 vaccines in the real world and a decline in personal vigilance were the main reasons for current vaccine hesitancy. A study demonstrated that vaccine hesitancy changes over time because of the ever-changing public’s perception of risk for COVID-19 and information related to the vaccination safety and efficacy [22]. As a result, our findings could assist relevant agencies in developing targeted strategies to continuously expand the acceptance and coverage of the third dose of COVID-19 vaccine.

Our findings indicated that 93.7% of participants were willing to receive a third dose of COVID-19 vaccines. Since China has a population of more than 1.4 billion, our results can help vaccine manufacturers in China to manage the vaccine production and distribution for the huge vaccine demand. Since vaccines produced in China need to be available not only to Chinese residents, but also to other countries struggling with this terrible pandemic. Meanwhile, at least 97% of the 3119 enrolled participants received at least one dose of COVID-19 vaccine, whether Sinopharm BIBP, Sinovac or Convidecia. This partly reflected a positive effect of vaccination history on receiving an additional dose. In a study consisting of 13,426 people from 19 countries/territories, 71.5% of participants indicated that they would be willing to accept the initial COVID-19 vaccine (not a third dose) if it was safe and efficacious [23]. A high degree of heterogeneity occurred in vaccine acceptance rates among different countries. Countries with an acceptance rate over 80% tended to be Asian countries with a high level of trust in government, and China ranked first with a rate of 88.6% [23]. The results of another vaccination willingness survey, assessed separately in China, also showed that 83.3% of 3541 valid responses showed a willingness to be vaccinated [20]. In addition, employer recommendations could be another positive factor in promoting vaccination [20,23]. Our results were consistent with this study, that is, civil servants working in government agencies or employees of public institutions had a higher willingness to be vaccinated than other occupational groups. Obviously, it may also have something to do with their access to more COVID-19 and vaccine-related knowledge.

The acceptance rate showed a unimodal change with increasing age. The highest acceptance rate was 94.6% (93.4–95.6%) in the 21–30 years aged group but decreased to 81.7% (72.3–88.9%) in people older than 50. Previous COVID-19 vaccine uptake surveys have shown higher uptake in older than younger groups, since older age acted as a risk factor for adverse outcomes of COVID-19 infection [23]. In Bangladesh, 71.3% of participants aged 51–60 years were willing to be vaccinated, while only 61.4% were willing to be vaccinated in the >60 years aged group [24]. Compared with the first two doses of vaccine, the single-peak change in the acceptance rate of an additional dose in the age group indicated that the middle-aged and elderly population had more concerns about the vaccine. Therefore, it would be advantageous to focus on this section of the population in the promotion of vaccination, and to emphasize health education on infection and vaccine-related knowledge.

In this study, the five dimensions of HBM provided a good framework for assessing participants’ attitudes towards a third dose of COVID-19 vaccination. In both univariable and multivariable analyses, most constructs in HBM models were significantly associated with vaccination intentions for a third dose of COVID-19. Our results are generally consistent with previous studies in China on the impact of health beliefs on vaccination acceptance [17,18,20]. Baccolini et al. found higher perceived COVID-19 severity was negatively associated with vaccine hesitancy (aOR = 0.89, 95% CI: 0.85–0.94) among Italian university students [22]. By contrast, higher susceptibility to COVID-19 did not show any relationship [22]. High cues to action were proved to have the most significant effect on vaccination willing-ness in our study [(cOR = 61.28, 95% CI: 32.17–116.72); (aOR = 23.66, 95% CI: 9.97–56.23)]. This also highlights the importance of health workers and community workers in the promotion of a third dose of COVID-19 vaccine. A total of 58.4% and 50.0% showed vaccination hesitation because they were unsure of its real-world effectiveness and safety, respectively. A systematic review indicated that one or two doses of COVID-19 vaccine were 85% (80–91%), 75% (71–79%), 54% (35–74%) and 74% (62–85%) effective against the alpha, beta, gamma, and delta variants, respectively. The overall pooled incidence of adverse events was 1.5% (1.4–1.6%) [25]. Another meta-analysis explained that the pooled frequency of vaccine-related serious adverse events was very low (<0.1%) and similar across all vaccine groups [26]. Among them, the incidence rate of adverse events reported by active surveillance was similar to clinical trial results, while it was lower in passive surveillance [26].

Studies into the safety and effectiveness of the third dose of COVID-19 vaccine continue to be published. A real-world study in Israel involving 1.15 million people found that a third dose of BNT162b2 vaccine was 91% effective against symptomatic infections, 93% effective against hospitalizations, and 92% effective against severe cases, compared with just two doses [8]. Among people over 60, the rate of confirmed infection was 11.3 (10.4–12.3) times lower in the third dose group, and the rate of severe disease was 19.5 (12.9–29.5) times lower than that in the initial two-dose group [27]. A prospective cohort study conducted from 17 February–30 June 2021, tested the safety and immunogenicity of the BNT162b2 vaccine as a third dose following two doses of the Sinopharm BBIP vaccine [28]. The GMT titer of anti-spike protein IgG antibody was increased from 1384 BAU/mL to 8040 BAU/mL after additional vaccination with the BNT162b2 vaccine, the incidence of adverse events was low, and most of them had a short-term appearance [28]. In addition, a third injection of Sinopharm BBIP not only effectively increased the proportion of memory B cells but also improved the affinity between RBD and memory B cells, and the protective effect was more effective and lasted longer [29]. Our findings are helpful to assess acceptance of the public for the third dose of COVID-19 vaccination and explore the factors influencing individual vaccination behavior, which could provide a basis for the design of subsequent immunization strategies and dissemination of relevant health education. There were also some limitations. First, it was a nationwide online survey, which might limit the representativeness of the study sample without Internet access. Then, medical workers and other occupational categories were not investigated separately, so it is impossible to conduct subgroup analysis for these specific subgroups. Separate surveys on this subject of health workers are supposed to be done in the future. Moreover, people’s acceptance of getting a third dose of COVID-19 vaccine was only measured by self-report, and we were unable to develop a standard scale to assess the willingness. Finally, we intend to focus on the willingness to receive the third dose of COVID-19 vaccine and its influencing factors, which cannot be equated with vaccination behavior.

It is strikingly noticeable that the current primary goal of immunization during the COVID-19 pandemic remains to prevent hospitalizations, severe illness, and death [9]. Thus, a third dose of COVID-19 vaccine may be needed only if there is evidence of inadequate protection against these adverse outcomes over time. The extent of population immunity declines, and the need for a third dose may vary from case to case, including different vaccine products, target populations, the prevalence of SARS-CoV-2 virus, especially significant SARS-CoV-2 variants and exposure intensity [9].

## 5. Conclusions

In this nationwide cross-sectional study, 93.7% of Chinese residents were willing to receive a third dose of COVID-19 vaccine, which can guide vaccine manufacturers in China to manage the vaccine production and distribution for the huge domestic and international vaccine demand. Acceptance behaviors were closely correlated with previous vaccination history, high level of perceived susceptibility and high level of action cues. Public doubts about the real-world safety and effectiveness of existing vaccines may lead to a part of hesitation. Therefore, our study can help the Chinese government and relevant agencies to develop more scientific and targeted roll-out strategies for the third dose of COVID-19 vaccine, which is essential at a time when the outbreak is still possible at any time. Of course, it also has certain reference significance for other countries to formulate vaccine promotion strategies.

## Figures and Tables

**Table 1:** Acceptance of a third dose of COVID-19 vaccine in China by demographic characteristics (n = 3119).

Characteristics	Number (%)	Acceptance of a Third Dose of COVID-19 Vaccination		*p* Value
		Yes (%)	95% CI	
**Sociodemographic characteristics**				
**Region**				0.06
Eastern	1357 (43.5)	1256 (92.6)	91.1–93.9	
Central	962 (30.8)	911 (94.7)	93.1–96.0	
Western	800 (25.6)	756 (94.5)	92.8–95.9	
**Age group(years)**				<0.01 *
≤20	162 (5.2)	150 (92.6)	87.8–95.9	
21–30	1747 (56.0)	1652 (94.6)	93.4–95.6	
31–40	934 (29.9)	877 (93.9)	92.2–95.3	
41–50	194 (6.2)	177 (91.2)	86.6–94.6	
>50	82 (2.6)	67 (81.7)	72.3–88.9	
**Sex**				0.08
Female	1608 (51.6)	1519 (94.5)	93.3–95.5	
Male	1511 (48.4)	1404 (92.9)	91.5–94.1	
**Education§**				<0.01 *
High school or polytechnic school	184 (5.9)	162 (88.0)	82.8–92.1	
Junior college	413 (13.2)	373 (90.3)	87.2–92.9	
Bachelor’s degree	2357 (75.6)	2238 (95.0)	94.0–95.8	
Postgraduate degree	165 (5.3)	150 (90.9)	85.8–94.6	
**Occupation**				<0.01 *
Civil servant	77 (2.5)	75 (97.4)	91.9–99.5	
Employees of enterprise	1986 (63.7)	1866 (94.0)	92.8–94.9	
Employees of public Institutions	367 (11.8)	359 (97.8)	95.9–99.0	
Individual household	238 (7.6)	208 (87.4)	82.7–91.2	
Student	384 (12.3)	361 (94.0)	91.3–96.1	
Others	67 (2.1)	54 (80.6)	70.0–88.7	
**Monthly household income per capita** **(RMB)**				0.01 *
≤3000	411 (13.2)	385 (93.7)	91.0–95.7	
3001–5000	558 (17.9)	506 (90.7)	88.1–92.9	
5001–10,000	1370 (43.9)	1285 (93.8)	92.4–95.0	
10,001–20,000	641 (20.6)	613 (95.6)	93.8–97.0	
>20,000	139 (4.5)	134 (96.4)	92.3–98.6	
**Health status**				
**History of chronic disease**				0.05
Yes	238 (7.6)	216 (90.8)	86.6–93.9	
No	2881 (92.4)	2707 (94.0)	93.0–94.8	
**History of COVID-19 vaccination**				<0.01 *
Sinopharm BIBP	1137 (36.5)	1085 (95.4)	94.1–96.5	
Sinovac	1770 (56.7)	1668 (94.2)	93.1–95.3	
Convidecia	118 (3.8)	110 (93.2)	87.6–96.7	
No	94 (3.0)	60 (63.8)	53.8–73.0	
**Knowledge factors**				
**Total knowledge score on COVID-19**				<0.01 *
Low (score 0–4)	26 (0.03)	19 (73.1)	54.3–87.1	
Moderate (score 5–10)	1495 (47.9)	1380 (92.3)	90.9–93.6	
High (score 11–15)	1598 (51.2)	1524 (95.4)	94.3–96.3	
**Total knowledge score on COVID-19 vaccination**				0.01 *
Low (score 0)	22 (0.71)	18 (81.8)	62.4–93.5	
Moderate (score 1–2)	1793 (57.5)	1665 (92.9)	91.6–94.0	
High (score 3)	1304 (41.8)	1240 (95.1)	93.8–96.2	
Total	3119 (100)	2923 (93.7)	92.9–94.6	

* *p* < 0.05. **§**Those with education degrees below high school were categorized into high schools or polytechnic schools due to its limited quantity. Sinopharm BIBP = Sinopharm COVID-19 vaccine from Beijing Institute of Biological Products Co., Ltd. (Beijing, China). Sinovac = Sinovac COVID-19 vaccine from Sinovac Life Sciences Co., Ltd. (Beijing, China). Convidecia = Recombinant COVID-19 vaccine (Adenovirus Type 5 Vector).

**Table 2:** Comparison of the acceptance of a third dose for COVID-19 vaccination based on the health belief model (n = 3119).

Dimensions of Health Belief Model	Item	Response	Number (%)	Acceptance of a Third Dose of COVID-19 Vaccine		*p* Value
				Yes (%)	95% CI	
Perceived susceptibility	Are you concerned about getting COVID-19	Not concerned	814 (26.1)	734 (90.2)	88.0–92.1	<0.05 *
		Concerned	2305 (73.9)	2189 (95.0)	94.0–95.8	
	Are you worried about your family contracting the COVID-19	Not concerned	547 (17.5)	488 (89.2)	86.4–91.6	<0.05 *
		Concerned	2572 (82.5)	2435 (94.7)	93.8–95.5	
Perceived severity	People who get COVID-19 are more likely to get severe illness	Disagree	1548 (49.6)	1438 (92.9)	91.5–94.1	0.06
		Agree	1571 (50.4)	1485 (94.5)	93.3–95.6	
	When you get COVID-19, your family’s health may be at risk	Disagree	192 (6.2)	164 (85.4)	79.9–89.9	<0.05 *
		Agree	2927 (93.8)	2759 (94.3)	93.4–95.1	
Perceived barriers	A third dose of COVID-19 vaccine can cause infection	Disagree	2903 (93.1)	2726 (93.9)	93.0–94.7	0.115
		Agree	216 (6.9)	197 (91.2)	86.9–94.4	
	It is not safe to get a third dose against COVID-19	Disagree	3079 (98.7)	2891 (93.9)	93.0–94.7	<0.05 *
		Agree	40 (1.3)	32 (80.0)	65.8–90.1	
	It is not effective to get a third dose against COVID-19	Disagree	2976 (95.4)	2811 (94.5)	93.6–95.2	<0.05 *
		Agree	143 (4.6)	112 (8.3)	71.0–84.5	
Perceived benefits	It is good to strengthen your health with COVID-19 vaccination	Disagree	794 (25.5)	678 (85.4)	82.8–87.7	<0.05 *
		Agree	2325 (74.5)	2245 (96.6)	95.8–97.2	
	It is good for family health when vaccinating a third dose	Disagree	801 (25.7)	685 (85.5)	83.0–87.8	<0.05 *
		Agree	2318 (74.3)	2238 (96.5)	95.7–97.2	
	A third dose can provide better protection against COVID-19	Disagree	479 (15.4)	400 (83.5)	80.0–86.6	<0.05 *
		Agree	2640 (84.6)	2523 (95.6)	94.7–96.3	
Cues to action	If your doctor/nurse recommends that you get a third dose against COVID-19, you will choose it	Disagree	509 (16.3)	404 (79.4)	75.7–82.7	<0.05 *
		Agree	2610 (83.7)	2519 (96.5)	95.8–97.2	
	If your family recommends you to get a third dose, you will take it	Disagree	709 (22.7)	600 (84.6)	81.8–87.1	<0.05 *
		Agree	2410 (77.3)	2323 (96.40)	95.6–97.1	
	If the community recommends that you get a third dose against COVID-19, you will choose it	Disagree	930 (29.8)	782 (84.10)	81.6–86.3	<0.05 *
		Agree	2189 (70.2)	2141 (97.80)	97.1–98.4	

* *p* < 0.05. “Very Concerned” and “Concerned” were combined to be “Concerned”; and “Disagree/Not Sure” were combined to be “Disagree”.

**Table 3:** Factors associated with the acceptance of a third dose of COVID-19 vaccine (n = 3119).

Characteristics	Univariable Model		Multivariable Model	
	Crude OR (95% CI)	*p* Value	Adjusted OR (95% CI)	*p* Value
**Sociodemographic characteristics**				
**Age group(years)**				
≤20	1 (reference)		1 (reference)	
21–30	1.39 (0.75–2.60)	0.30	1.22 (0.50–2.98)	0.67
31–40	1.23 (0.65–2.35)	0.53	1.19 (0.45–3.19)	0.73
41–50	0.83 (0.39–1.80)	0.64	1.15 (0.37–3.58)	0.81
>50	0.36 (0.16–0.81)	0.01 *	0.39 (0.12–1.33)	0.13
**Education§**				
High school or polytechnic school	1 (reference)		1 (reference)	
Junior college	1.27 (0.73–2.20)	0.40	0.88 (0.42–1.81)	0.72
Bachelor’s degree	2.55 (1.58–4.14)	<0.01 *	1.20 (0.60–2.39)	0.61
Postgraduate degree	1.36 (0.68–2.72)	0.38	0.61 (0.23–1.65)	0.33
**Occupation**				
Civil servant	1 (reference)		1 (reference)	
Employees of enterprise	0.42 (0.10–1.71)	0.22	0.21 (0.046–0.98)	0.05 *
Employees of public Institutions	1.20 (0.25–5.745)	0.82	0.84 (0.15–4.59)	0.84
Individual household	0.19 (0.04–0.79)	0.02 *	0.11 (0.02–0.53)	0.01 *
Student	0.42 (0.10–1.81)	0.24	0.18 (0.03–1.01)	0.05
Others	0.11 (0.02–0.51)	0.01 *	0.10 (0.02–0.57)	0.01 *
**Monthly household income per capita (RMB)**				
≤3000	1 (reference)		1 (reference)	
3001–5000	0.66 (0.40–1.07)	0.09	0.69 (0.31–1.54)	0.37
5001–10,000	1.02 (0.65–1.61)	0.93	0.72 (0.32–1.65)	0.44
10,001–20,000	1.48 (0.85–2.56)	0.16	1.25 (0.50–3.13)	0.64
>20,000	1.81 (0.68–4.81)	0.23	2.13 (0.55–8.28)	0.27
**History of COVID-19 vaccination**				
Sinopharm BBIP	11.82 (7.14–19.58)	<0.01 *	6.55 (3.30–12.98)	<0.01 *
Sinovac	9.27 (5.82–14.77)	<0.01 *	5.22 (2.72–10.02)	<0.01 *
Convidecia	7.79 (3.39–17.90)	<0.01 *	5.80 (2.04–16.48)	<0.01 *
No	1 (reference)		1 (reference)	
Knowledge factors				
**Total knowledge score on COVID-19**				
Low (score 0–4)	1 (reference)		1 (reference)	
Moderate (score 5–10)	4.42 (1.82–10.74)	<0.01 *	0.60 (0.17–2.12)	0.42
High (score 11–15)	7.59 (3.09–18.61)	<0.01 *	0.89 (0.24–3.21)	0.85
**Total knowledge score on COVID-19 vaccination**				
Low (score 0)	1 (reference)		1 (reference)	
Moderate (score 1–2)	2.89 (0.96–8.67)	0.06	0.41 (0.10–1.76)	0.23
High (score 3)	4.31 (1.42–13.09)	0.01 *	0.49 (0.11–2.12)	0.34
**Health belief factors**				
**Perceived susceptibility**				
Low	1 (reference)		1 (reference)	
Moderate	1.89 (1.38–2.59)	<0.01 *	2.41 (1.62–3.58)	<0.01 *
High	2.60 (1.63–4.13)	<0.01 *	2.48 (1.42–4.31)	<0.01 *
**Perceived severity**				
Low	1 (reference)		1 (reference)	
Moderate	1.00 (0.43–2.35)	1.00	0.45 (0.14–1.46)	0.18
High	1.54 (0.65–3.65)	0.33	0.48 (0.15–1.56)	0.22
**Perceived barriers**				
Low	5.40 (2.34–12.45)	<0.01 *	2.706 (0.92–7.93)	0.07
Moderate	0.87 (0.38–2.01)	0.75	0.25 (0.16–0.37)	0.49
High	1 (reference)		1 (reference)	
**Perceived benefit**				
Low	1 (reference)		1 (reference)	
Moderate	3.45 (1.57–7.57)	<0.01 *	0.50 (0.16–1.55)	0.23
High	19.17 (8.70–42.26)	<0.01 *	1.78 (0.56–5.66)	0.33
**Cues to action**				
Low	1 (reference)		1 (reference)	
Moderate	7.30 (3.90–13.67)	<0.01 *	3.91 (1.68–9.10)	<0.01 *
High	61.28 (32.17–116.72)	<0.01 *	23.66 (9.97–56.23)	<0.01 *

* *p* < 0.05. **§**Those with education degrees below high school were categorized into high schools or polytechnic schools due to its limited quantity. Sinopharm BIBP = Sinopharm COVID-19 vaccine from Beijing Institute of Biological Products Co., Ltd. (Beijing, China). Sinovac = Sinovac COVID-19 vaccine from Sinovac Life Sciences Co., Ltd. (Beijing, China). Convidecia = Recombinant COVID-19 vaccine (Adenovirus Type 5 Vector).

## Data Availability

All data in the study are available from the corresponding author by request.

## Supplementary Materials

The following are available online at https://www.mdpi.com/article/10.3390/vaccines10010089/s1, Table S1 Collection of valid questionnaires by provinces in mainland China.
