# Supplementary material for: Acceptance of a Third Dose of COVID-19 Vaccine and Associated Factors in China Based on Health Belief Model: A National Cross-Sectional Study
Source: Vaccines (Basel). 2022 Jan 7;10(1):89. doi: 10.3390/vaccines10010089 (PMC8780099; doi:10.3390/vaccines10010089)
Supplement: Supplementary file 1 [file vaccines-10-00089-s001.zip › vaccines-1505570-supplementary.pdf]

**Supplemental file**

**Table S1** Collection of valid questionnaires by region in mainland China

| Regions | Number | Proportion |
|---|---|---|
| Guangdong Province | 272 | 8.72% |
| Shandong Province | 233 | 7.47% |
| Henan Province | 228 | 7.31% |
| Jiangsu Province | 186 | 5.96% |
| Sichuan Province | 186 | 5.96% |
| Hebei Province | 168 | 5.39% |
| Hunan Province | 151 | 4.84% |
| Zhejiang Province | 147 | 4.71% |
| Anhui Province | 140 | 4.49% |
| Hubei Province | 132 | 4.23% |
| Guangxi Province | 116 | 3.72% |
| Jiangxi Province | 105 | 3.37% |
| Liaoning Province | 96 | 3.08% |
| Fujian Province | 95 | 3.05% |
| Shaanxi Province | 92 | 2.95% |
| Guizhou Province | 88 | 2.82% |
| Shanxi Province | 76 | 2.44% |
| Chongqing | 74 | 2.37% |
| Heilongjiang Province | 74 | 2.37% |
| Yunnan Province | 66 | 2.12% |
| the Nei Monggol Autonomous Region | 57 | 1.83% |
| Jilin Province | 56 | 1.80% |
| Shanghai | 55 | 1.76% |
| Gansu Province | 51 | 1.64% |
| Beijing | 49 | 1.57% |
| the Xinjiang Uygur Autonomous Region | 41 | 1.31% |
| Tianjin | 31 | 0.99% |
| Hainan Province | 25 | 0.80% |
| Ningxia Province | 18 | 0.58% |
| Tibet | 7 | 0.22% |
| Qinghai Province | 4 | 0.13% |
